# Cell death mechanisms in head and neck cancer cells in response to low and high-LET radiation

**DOI:** 10.1017/erm.2021.31

**Published:** 2022-01-12

**Authors:** Maria Rita Fabbrizi, Jason L. Parsons

**Affiliations:** 1 Department of Molecular and Clinical Cancer Medicine, Cancer Research Centre, University of Liverpool, 200 London Road, Liverpool, L3 9TA, UK; 2 Clatterbridge Cancer Centre NHS Foundation Trust, Clatterbridge Road, Bebington, CH63 4JY, UK

**Keywords:** Apoptosis, autophagy, cell death, ionising radiation, linear energy transfer, proton beam therapy, radiotherapy, senescence

## Abstract

Head and neck squamous cell carcinoma (HNSCC) is a common malignancy that develops in or around the throat, larynx, nose, sinuses and mouth, and is mostly treated with a combination of chemo- and radiotherapy (RT). The main goal of RT is to kill enough of the cancer cell population, whilst preserving the surrounding normal and healthy tissue. The mechanisms by which conventional photon RT achieves this have been extensively studied over several decades, but little is known about the cell death pathways that are activated in response to RT of increasing linear energy transfer (LET), including proton beam therapy and heavy ions. Here, we provide an up-to-date review on the observed radiobiological effects of low- versus high-LET RT in HNSCC cell models, particularly in the context of specific cell death mechanisms, including apoptosis, necrosis, autophagy, senescence and mitotic death. We also detail some of the current therapeutic strategies targeting cell death pathways that have been investigated to enhance the radiosensitivity of HNSCC cells in response to RT, including those that may present with clinical opportunities for eventual patient benefit.

## Introduction

**Fig. 1.**
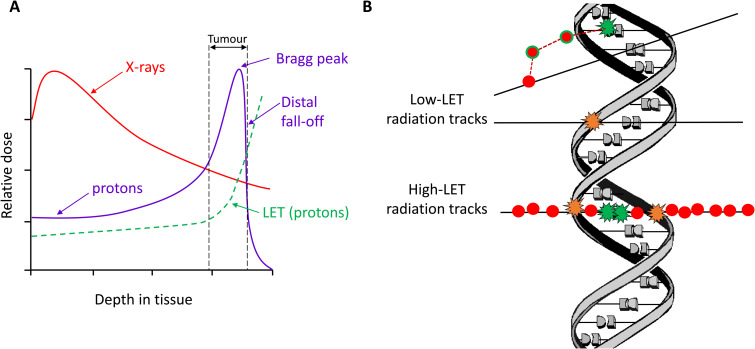
Depth-dose distribution of x-rays (photons) versus protons and relationship to LET leading to DNA damage. (a) Comparison of the dose delivered related to depth in tissue of photons versus protons. Proton irradiation, unlike photons, leads to targeted delivery of the radiation dose to the tumour thus minimising associated normal tissue irradiation, but which leads to associated increases in LET at and around the Bragg peak. (b) Tracks of IR of different LET and their interaction with DNA. Ionisation events (red dots) can occur indirectly (predominant with low-LET radiation) or directly (particularly with high-LET radiation) leading to DNA damage in the form of strand breaks and base damage (orange and green stars, respectively). The low-LET radiation tracks generate largely isolated DNA damage, whereas the densely ionising tracks of high-LET radiation lead to significant levels and formation of CDD.

Head and neck squamous cell carcinoma (HNSCC) is the eighth most common cancer in the UK, with more than 12 000 new cases every year and a one-year survival rate as low as 20% in the hypopharyngeal cancer subtype (Ref. 1). The common risk factors associated with HNSCC are tobacco and alcohol consumption and infection by high-risk (type-16/18) human papillomaviruses (HPV). Interestingly, patients with HPV-positive HNSCC are known to have a better prognosis and improved survival rates due to their improved response to radiotherapy (RT) and chemotherapy as compared with HPV-negative HNSCC (Refs 2, 3). Several *in vitro* studies conducted using HNSCC cell lines have investigated the different molecular mechanisms and biological characteristics responsible for the increased radiosensitivity of HPV-positive cells, and have identified an association with defects in the efficiency of DNA double-strand break repair (reviewed in (Ref. 4)). Treatments include surgery, chemotherapy and RT (ionising radiation; IR), where mostly conventional photon (X-ray) radiation is used. However in recent years, use of proton beam therapy (PBT) has increased and has shown significant improvement in HNSCC treatment (Ref. 5). This is due to the fact that PBT reduces the degree of healthy tissue injury compared to photon irradiation, due to the characteristic low entrance dose and high in-depth energy deposition at a narrow and well-defined range called the Bragg peak (Fig. 1a). Moreover, X-rays are a low linear energy transfer (LET) radiation treatment which yields a reduced energy deposition along the path of the beam and a lower ionisation density resulting in spatially separated damage to vital macromolecules, particularly DNA (Fig. 1b). In comparison, PBT displays increases in LET at the Bragg peak and beyond the distal edge, which creates ionisation events and damage that is in closer proximity, such as the induction of complex DNA damage (CDD) containing multiple DNA lesions (Ref. 6). This increase in CDD represents a challenge to the cellular DNA repair machinery and therefore can contribute to the therapeutic effect of PBT, and more so of heavy ions (such as carbon) that are of significantly higher LET.

**Fig. 2.**
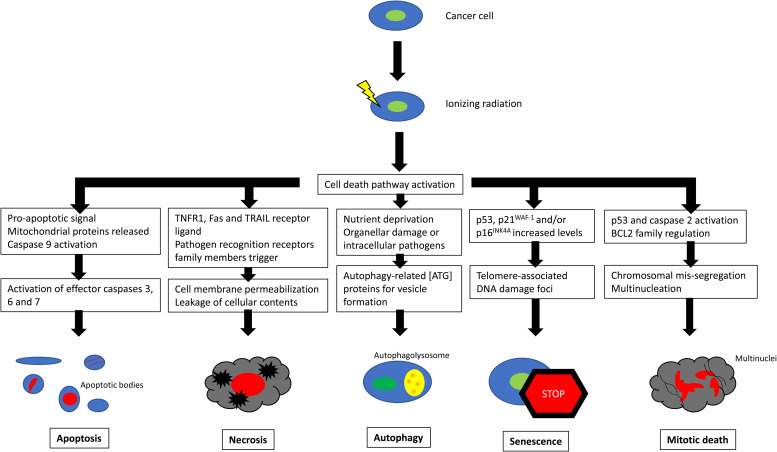
Cell death pathways responsive to IR. Depending on the level of DNA damage and cell type, one of the pathways including apoptosis, necrosis, autophagy, senescence and mitotic death will be initiated. The key steps and proteins involved in coordinating these pathways are shown. If the cell undergoes an initiation cell death pathway (senescence and mitotic catastrophe), then an executive pathway (apoptosis, necrosis and autophagy) will follow eventually.

Nevertheless, and independently from the source and type of IR used, the main goal of RT is to cause sufficient damage to macromolecules particularly DNA, but also to lipids, proteins and many metabolites and therefore to promote cancer cell death while preserving the surrounding healthy tissue. The latter is where targeted dose delivery and energy deposition by PBT have a significant advantage over conventional photon irradiation. Despite this, there are several mechanisms of cell death that may account for IR-induced cell killing, namely apoptosis (Refs 7, 8), necrosis (Ref. 8), mitotic catastrophe (Ref. 9), senescence (Ref. 9) and autophagy (Ref. 10) (Fig. 2). The mechanisms by which photon (X-ray) irradiation kills cancer cells have been studied in depth (Refs 11–13), but little is known in relation to PBT and other high-LET particles, including carbon ions. In this review, we will provide details on the different cell death mechanisms and the key proteins driving these responses, but then focus on exploring the cellular pathways reportedly involved in IR-induced cell killing particularly in HNSCC cells, highlighting any reported differences between low- and high-LET radiation. Finally, we will present the therapeutic strategies available within these cell death mechanisms that are currently considered to enhance cancer cell radiosensitivity, and which have the potential to move forward into the clinic to improve HNSCC treatment.

## Initiating and executive mechanisms of cell death

Cell death is a natural consequence of the life cycle progression, although it can also occur if a cell becomes redundant, damaged beyond repair or harmful for the organism (Ref. 14). The mechanism chosen by the cell to die is dependent on the type and the extent of damage, but any cellular death pathway shows specific morphological alterations. For example, apoptosis shows cytoplasmic shrinkage, chromatin condensation and nuclear fragmentation resulting finally with the formation of small vesicles or apoptotic bodies released from the cells which are phagocytosed by the surrounding cells. Autophagy includes cytoplasmic vacuolisation and similarly culminates in phagocytic uptake, while necrotic cells exhibit loss of cytoplasm and damaged nuclear membranes (Ref. 15). Intracellular vacuolisation, cellular/nuclear enlargement and altered chromatin structure are usually observed in senescent cells, while nuclear changes due to chromosomal mis-segregation and/or persistence of acentric chromosomes, such as multinucleation, are commonly seen in cells undergoing mitotic catastrophe (Ref. 16). Apoptosis, necrosis and autophagy can be considered as executive mechanisms of cell death, while senescence and mitotic catastrophe should be categorised as initiating as these processes do not cause cell death themselves but only act as a trigger of another cell death pathway. Several other cell death mechanisms have been observed, including necroptosis and ferroptosis (Refs 17–20), but for the purpose of this review we will focus on those listed above.

### Apoptosis

**Fig. 3.**
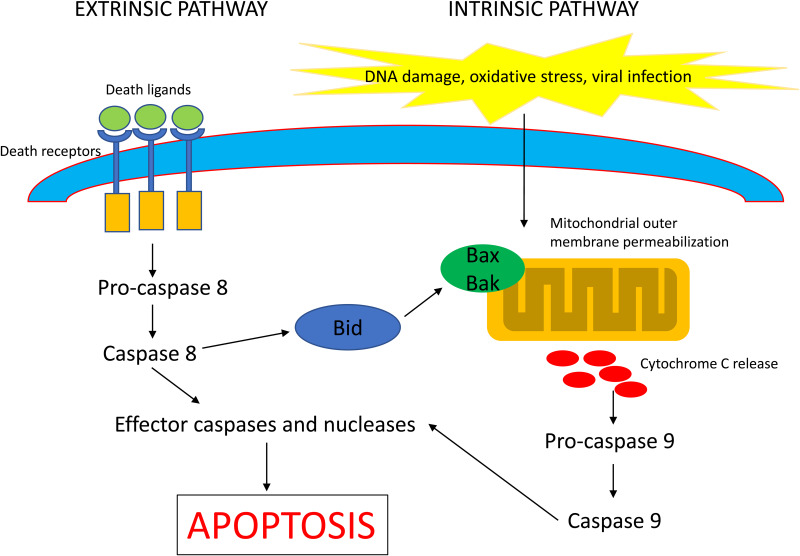
The extrinsic and intrinsic pathways of apoptosis. The extrinsic death receptor pathway is activated by death receptor ligands, including FasL, TNF-*α* or TRAIL, which in turn activates caspase 8 and downstream executing caspases. The intrinsic death receptor pathway is initiated by several intracellular stresses, leading to activation of Bax and Bak on the mitochondrial membrane and which result in the release of cytochrome c from the mitochondria. Cytoplasmic cytochrome c activates caspase 9 and downstream executing caspases.

Apoptosis is a form of programmed cell death characterised by specific morphological changes (Ref. 21), which consists of two major subtypes, namely the extrinsic and intrinsic apoptotic pathways (Fig. 3). Extrinsic apoptosis is mediated by membrane receptors, especially by death receptors (e.g. Fas cell surface death receptor and tumour necrosis factor (TNF) receptor superfamily member), and is driven mostly by the initiator caspases 8 and 10 (Ref. 22). Initiator caspase 9 can also trigger extrinsic apoptosis together with unc-5 netrin receptor B and DCC netrin 1 receptor (Ref. 23), although this is mostly involved in the activation of the intrinsic apoptotic pathway. Intrinsic apoptosis starts with mitochondrial outer membrane permeabilisation which is controlled via a fine balance of BCL2 family pro- and anti-apoptotic members, including BCL2-associated X, apoptosis regulator (BAX), BCL2 antagonist/killer 1 (BAK1) and BCL2 and BCL2-like 1 (BCL2L1) (Refs 24, 25). When the pro-apoptotic signal overcomes the anti-apoptotic one, mitochondrial proteins are released into the cytoplasm (e.g. cytochrome C and diablo IAP-binding mitochondrial protein) and this triggers initiator caspase 9 activation (Ref. 26). Both the intrinsic and extrinsic pathways of apoptosis proceed with the activation of effector caspases (caspases 3, 6 and 7), which in turn catalyse the specific cleavage of many key cellular proteins. Other members of the cysteine-dependent aspartate-specific protease family are caspase 2 (initiator caspase), caspases 1, 4, 5, 11 and 12 (inflammatory caspases) and caspase 14 (keratinisation-relevant caspase). In terms of morphological features, apoptotic cells show chromatin condensation which progresses into nuclear fragmentation as the apoptotic process proceeds, and this ends with the formation of apoptotic bodies and phagocytosis by the surrounding cells (Ref. 21).

### Necrosis

Necrosis is usually induced by several physical or chemical stress factors, including ischemia and hypoxia. The main event in necrosis is mitochondrial inner membrane depolarisation and outer mitochondrial membrane rupture, due mostly as a consequence of an increase in Ca^2+^ ions, ATP depletion and reactive oxygen species production (Ref. 27). However, other metabolic changes have been observed in cells undergoing necrotic death (Ref. 28). Necrotic cell death can also be induced when ligands bind to specific receptors, such as TNF receptor 1, Fas and TRAIL receptor, although these activation pathways are equally shared within apoptosis (Refs 29, 30). Necrosis is also induced by biological stress triggers such as external pathogens, which are recognised by pathogen recognition receptor family members, including the membrane-associated Toll-like receptors, the cytosolic NOD-like receptors and RIG-I-like receptors (Refs 31–33). At a macroscopic level, necrosis stimulates the cell membrane to become permeable early in the process, followed by leakage of the cellular contents. In the case of autophagic-like necrosis, numerous vacuoles are observed in the cytoplasm, while dilation of organelles and empty spaces is detectable if the cell undergoes non-lysosomal-type necrosis (Ref. 34).

### Autophagy

**Fig. 4.**
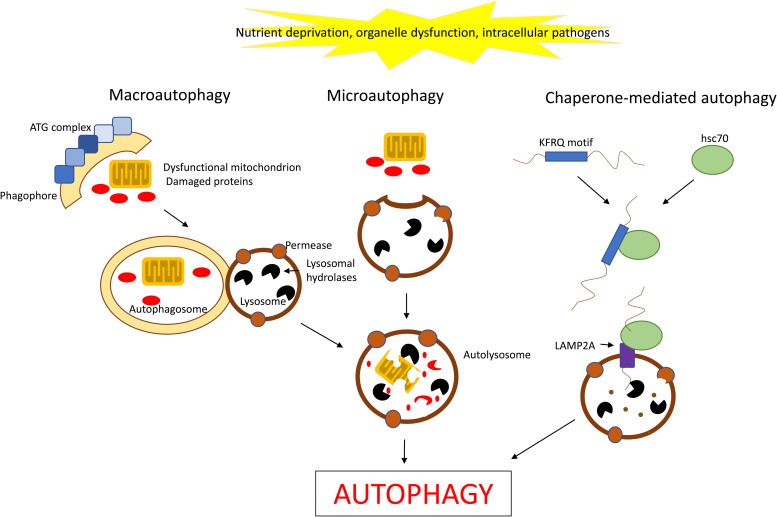
The mechanisms of autophagy. Schematic representation of the three main autophagy pathways: macro-, micro- and chaperone-mediated autophagy. Macroautophagy sequesters cytosolic cargo inside a phagophore formed by specific ATG proteins and lipids. The membrane then seals into an autophagosome and fuses with lysosomes causing the degradation of the trapped cargo. Microautophagy entraps cytosolic cargo in small vesicles formed by invagination of the lysosomal membrane. Chaperone-mediated autophagy involves the selective degradation of KFRQ-like motif-bearing proteins delivered to the lysosomes via chaperone HSC70 and their internalisation in lysosomes via the receptor lysosome-associated membrane protein type 2A (LAMP2A).

Autophagy is a highly regulated mechanism by which the cell removes unnecessary or dysfunctional components by self-degradation and recycling within the cell. Autophagy can be categorised into three forms: macroautophagy (or simply autophagy), microautophagy and chaperone-mediated autophagy (Fig. 4). Macroautophagy requires an intermediate single-membrane vesicle called a phagophore that engulfs the cytoplasm containing the components to be degraded, and rounds up becoming a double-membrane autophagosome. This will then fuse with an endosome or lysosome and releases its contents into the lytic organelle for degradation. Microautophagy, on the other hand, does not have any intermediates and the cellular content to be removed is directly engulfed by an endosome or lysosome (Ref. 35). Chaperone-mediated autophagy requires firstly the recognition of a specific motif in the protein to be degraded by heat shock cognate 71 kDa protein (hsc70), which will then bind the cytosolic tail of lysosome-associated membrane protein type 2A on the lysosomal membrane and transport the substrate into the lysosomal lumen where it will be rapidly degraded (Ref. 36). The proteins involved in vesicle formation can be grouped together as the autophagy-related (ATG) proteins, which collectively provide a very dedicated and fine machinery (Ref. 37). Autophagic responses are usually triggered by nutrient deprivation, but also by organelle damage or by intracellular pathogens (Ref. 38). Since autophagy may act as a tumour suppressor, an impaired mechanism could lead to the accumulation of toxic proteins and organelles, such as dysfunctional mitochondria, thus promoting oxidative stress, accumulation of DNA lesions and genomic instability, ultimately leading to promotion of cancer transformation (Ref. 39). Recently, it has been suggested that basic autophagy can actually maintain the survival of cancer cells in their unique environment (Ref. 40).

### Senescence

The term ‘replicative cellular senescence’ indicates a process in which cells are alive but are unable to undergo further cell division and therefore are in a permanent state of growth arrest (Ref. 41). Senescence can be triggered by a variety of factors and is usually associated with increased levels of p53, p21^WAF–1^ and/or p16^INK4A^, but telomere-associated DNA damage foci are also often present (Ref. 42). The molecular mechanisms involved in senescence pathway activation and execution have been extensively studied and reviewed (Refs 41, 43). Assessment of senescent cells can be easily performed with histochemical staining of *β*-galactosidase (Refs 44, 45), although other markers of senescence that have been identified include Cathepsin D and Dec-1 (Refs 46, 47). Morphological changes in senescent cells include increased granularity and cytoplasmic vacuolar content, followed by cell flattening. Senescence has always been considered an irreversible process, due to the major metabolic modifications the cell undergoes together with genetic and structural alterations, although several studies have highlighted the possibility of cells escaping senescence. In fact, in the case of senescence induced by chemotherapy or RT, several studies have reported that cells were able to re-enter the cell cycle due to overexpression of the cyclin-dependent kinase, Cdc2/Cdk1 or its downstream target survivin (Refs 48–50). Considering that most tumours have inactive p53 and/or p16INK4A/Rb signalling pathways, escaping senescence should be considered as a possibility.

### Mitotic catastrophe

The term ‘mitotic catastrophe’, or the more appropriate phrase ‘mitotic death’, indicates cell death induced by aberrant mitosis (Ref. 17). Cells undergoing mitotic catastrophe show unique morphologically defined features, such as multinucleation and micronucleation, mainly due to the chromosomal mis-segregation characteristic of this process (Ref. 16). Mitotic death can be triggered by either exogenous or endogenous sources, which ultimately cause several cell dysfunctions, such as altered DNA replication and chromosome segregation, and interference with microtubular dynamics (Refs 51–53). The molecular mechanisms involved in mitotic catastrophe are still under investigation, although p53 seems to play a role in the trigger signal (Ref. 16) and to activate the transduction cascade via caspase 2, which in turn initiates a variant of intrinsic apoptosis regulated by members of the BCL2 protein family (Refs 54, 55). However, many cancer cell types lack functional p53 and in those cases it appears that cells showing massive chromosomal aberration are driven preferentially into necrosis (Ref. 56). It is interesting to note that cell death is not the only possible fate aberrant mitotic cells can face, as it has been observed that cells escaping the mitotic block can instead enter p53-mediated or hippo-mediated cellular senescence (Refs 57–60), or survive as polyploid and aneuploid cells and initiate neoplastic transformation and progression (Ref. 16). However, a recent study suggests that cells escaping mitotic catastrophe stimulate the immunological response which in turn will trigger cell degradation (Ref. 61), providing an efficient mechanism for the control of tissue homeostasis.

## Radiation-induced cell death mechanisms

Generally, the therapeutic effect of RT is achieved through sufficient cell injury, particularly in terms of macromolecule and DNA damage, to overcome the cancer cells ability to repair the damage and therefore forcing the cell into initiating a cell death-activated pathway. The form of cell death induced by a particular anti-cancer agent such as IR depends on several factors, including cell type, the type of DNA damage to which the cell is exposed and the dose of the agent used (Refs 56, 62). For example, *γ*-radiation exposure can cause a massive apoptotic response in T and B cells but not in monocyte-derived macrophages and immature dendritic cells *ex vivo* (Ref. 63). Furthermore, it is known that X-rays and PBT cause a different spectrum of DNA damage in cancer cells due to changes in energy/LET (Refs 6, 64), which is likely to trigger a different cell death response. Therefore, whilst low-LET X-rays generally induce a high proportion of DNA base damage and single DNA strand breaks (SSB) relative to DNA double-strand breaks (DSB), higher LET radiation exposure including PBT but more so heavy ions can induce increased amounts and complexity of CDD. CDD is defined as multiple DNA damage types within close proximity (1–2 helical turns of the DNA), and can be classified as either DSB-associated or non-DSB-associated (Ref. 65). This though suggests that depending on the spectrum of DNA damage induced, different radiation sources can cause different cell death mechanisms to be activated that should be considered. Furthermore in addition to the type of damage, the dose of IR leading to a specific level of DNA damage may also have a major influence on the selection of cell death mechanism triggered, as well as the switching between these events given that many cell death mechanisms share several initiating factors. Finally, the long held concept that cells either repair their damage or undergo apoptosis after IR treatment is outdated, and the role of apoptosis in the tumour response to radiation has been minimised considering that most tumours actually lose the ability to initiate the apoptotic pathway (Ref. 66). A more important role in the anti-tumour effect of radiation is played by mitotic catastrophe or senescence, although as already stated previously, both these mechanisms cannot be considered strictly cell death and therefore rely on other pathways (e.g. apoptosis or autophagy) that trigger this phenotype.

### Cell death mechanisms after low LET exposure

As mentioned above, tumour cells post-IR can undergo apoptosis, although recent studies have implicated a reduced contribution of this particular pathway to the total amount of cell death to a relatively low level (Refs 67, 68). Apoptosis detection in several tumour cell lines, including breast cancer, non-small-cell lung cancer and colorectal cancer, has been reported to never exceed 30% of the total even at significantly high doses of radiation (Ref. 69). HNSCC cell lines appear to show no difference in the levels of IR-induced apoptosis, with the vast majority of the studies agreeing that low-LET radiation (e.g. X-rays and *γ*-rays) does not cause significant apoptosis activation (summarised in Table 1). In fact, thyroid cancer cells show no apoptotic response after a relatively low dose of 3 Gy (Ref. 70), and only a modest increase after 20 Gy treatment (Ref. 71), whilst laryngeal squamous carcinoma cells appear to reach an increase in 20% of apoptotic cells after a fractionated 10 Gy dose (Ref. 72). The HNSCC cell lines UM-SCC1, UM-SCC6 (both HPV-negative) and UPCI-SCC-154 (HPV-positive) showed no difference in caspase 3 activation and Annexin V detection at 2 and 4 Gy X-ray exposure (Refs 73, 74). Similarly in nasopharyngeal carcinoma cell lines, no difference in apoptosis was observed after 8 Gy (Ref. 75), although in contrast another study showed a 10-fold increase in the apoptotic response after 10 Gy (Ref. 76).

**Table 1.** Biological effects of low-LET radiation on head and neck cancer cells

Cancer cell	Dose^a^	Response compared to control	Reference
Thyroid cancer cells	3 Gy	No apoptotic response	(Ref. 70)
	20 Gy	Modest apoptotic response	(Ref. 71)
Laryngeal carcinoma cells	Fractionated 10 Gy	20% increase in apoptotic cells	(Ref. 72)
UM-SCC1 (oral cavity), UM-SCC6 (oropharynx) UPCI-SCC-154 (oral cavity)	2 and 4 Gy	No caspase 3 activation	(Refs 73, 74)
Nasopharyngeal carcinoma cells	8 Gy	No apoptotic response	(Ref. 75)
Nasopharyngeal carcinoma cells	4 Gy	20% increase in apoptotic cells	(Ref. 76)
SqCC/Y1 (oral cavity)	4 Gy	Higher necrotic response	(Ref. 77)
HN5 (oral cavity)	4 Gy	No increase in necrotic cells	(Ref. 77)
UPCI-SCC-154 (oral cavity)	4 Gy	No increase in necrotic cells	(Ref. 77)
UM-SCC6 (oropharynx)	4 Gy	No increase in necrotic cells	(Ref. 77)
Nasopharyngeal carcinoma cells	4 Gy	Increase in the ratio of LC3II/LC3I	(Ref. 78)
SQ20B (larynx)	4 Gy	No increase in several autophagy-related proteins	(Ref. 79)
Cal-33 (oral cavity)	6 Gy	2 to 5-fold increase in several autophagy-related proteins	(Ref. 80)
SCC61 (oral cavity)	10 Gy *γ*-rays	76% of senescence-induced cells	(Ref. 81)
SQ20B (larynx)	10 Gy *γ*-rays	18% of senescence-induced cells	(Ref. 81)
SqCC/Y1 (oral cavity)	4 Gy	Increased percentage of senescent cells	(Ref. 77)
HN5 (oral cavity)	4 Gy	Increased percentage of senescent cells	(Ref. 77)
UPCI-SCC-154 (oral cavity)	4 Gy	Increased percentage of senescent cells	(Ref. 77)
UM-SCC6 (oropharynx)	4 Gy	Increased percentage of senescent cells	(Ref. 77)
Detroit 562 (pharynx)	2 Gy	No increase in senescent cells	(Ref. 82)
Nasopharyngeal carcinoma cells	6–10 Gy	56 and 79% IR-induced cellular senescence	(Ref. 83)
FaDu (hypopharynx)	6 Gy	20% cells undergo mitotic catastrophe	(Ref. 84)
Oesophageal carcinoma cells	6 Gy	Modest increase in mitotic catastrophe	(Ref. 85)
Nasopharyngeal carcinoma cells	20 Gy	50% of cells undergo mitotic catastrophe	(Ref. 86)

a X-ray radiation treatment unless differently stated.

Necrosis pathway activation has mostly been considered more as a side-effect of RT in surrounding healthy tissue, rather than a cell death mechanism for cancer cells. Patients undergoing RT for HNSCC can experience significant side-effects, including osteoradionecrosis and oral cavity necrosis, which may require surgical intervention (Ref. 87). The mechanism underlying this specific necrotic transformation is not fully understood, although fibrosis may play a major role. This is due to the fact that RT increases the levels of reactive oxygen species-mediated cytokines, such as TNF-*α*, transforming growth factor-*β*1 and connective tissue growth factor, resulting in unregulated fibroblastic activation (Ref. 88). Human and mouse leukaemia cells have been shown to undergo necrosis after 300 and 9 Gy X-ray exposure, respectively (Refs 89, 90), suggesting the radiosensitive nature of the murine cells. HNSCC cells appear to undergo necrosis after X-ray radiation exposure at different degrees depending on cell type and HPV status (summarised in Table 1). In fact 48 h after irradiation, HPV-negative SqCC/Y1 cells showed a significantly higher percentage of necrotic cells compared to the unirradiated controls, but this was not observed in another HPV-negative cell line (HN5) or in two HPV-positive cell lines (UPCI-SCC-154 and UMSCC-47) tested (Ref. 77). One explanation, at least in the HPV-positive HNSCC cell lines, could be provided by the fact that the HPV oncoprotein E7 inhibits necrosis activation, thus making these cell lines less prone to this mechanism of cell death (Ref. 91).

Since autophagy is mostly associated with tumour suppression, increases in ATG proteins are to be expected following RT, and have been observed in HNSCC (summarised in Table 1). The ratio of LC3II/LC3I (an autophagic marker) has been shown to increase significantly in nasopharyngeal carcinoma cells after exposure to 4 Gy X-rays (Ref. 78), and protein level analysis showed that autophagic signalling proteins including Beclin1, Atg5, Atg7 and LC3B were upregulated in a time- and dose-dependent manner post-irradiation (Ref. 92). However, the same treatment did not appear to trigger any LC3B activation in SQ20B carcinoma cells (Ref. 79), suggesting a role for a differential gene expression profile in contributing to autophagy specific for each cell line. Exposure to 6 Gy of low-LET X-rays radiation caused a 2–5-fold increase in several ATG proteins in Cal-33 carcinoma cells, including LC3B, p62, Atg4A and Atg4B (Ref. 80). However, although autophagy directly contributes to death in stressed cells (Ref. 93), several studies have actually suggested a protective role for autophagy, which actually helps cancer cells to survive by reducing cell damage (Ref. 94). Autophagy is frequently activated in radioresistant cancer cells (Ref. 95) and the pathway shares upstream mediators with apoptosis, indicating a clear cross-talk between the two mechanisms and a fine balance between cell death and cell survival in response to IR. It has been proposed, in fact, that low-energy X-rays trigger the NF-*κ*B pathway and induce Beclin1 gene expression, which consequently activates autophagy and confers radioresistance in HNSCC cancer cells (Ref. 96). Moreover, apoptosis can be inhibited by autophagy proteins, such as Beclin1, which can degrade caspase 8 and interfere with the activation of Bid (Ref. 97), reducing cell death levels after irradiation (Ref. 96).

Senescent cells induced by RT usually trigger inflammation, which in turn leads to the targeted removal of cancer cells (Ref. 98), making senescence an outcome at the initial treatment stages. In contrast, some studies suggest the possibility of senescence being a protective mechanism for cancer cells, causing increased aggressiveness and metastasis (Refs 99, 100). Relatively radiosensitive SCC61 HNSCC cells were demonstrated to accumulate 76% of senescent cells, while radioresistant SQ20B cells only displayed 18% of cells positive for senescence markers after 10 Gy *γ*-ray exposure (Ref. 81) (summarised in Table 1). X-ray (4 Gy) treatment significantly increased the percentages of senescent cells at 4 and 6 days post-IR in two HPV-negative HNSCC cell lines (SqCC/Y1 and HN5) and two HPV-positive cell lines (UPCI-SCC-154 and UMSCC-47), although the absolute percentage only ranged from 5 to 18% (Ref. 77). In contrast, Detroit562 HNSCC cells (HPV-negative) showed no increase in the levels of senescence markers, such as p21 and *β*-galactosidase, after 2 Gy X-rays (Ref. 82). The potentially higher level of senescent cells present following irradiation of HPV-positive cells can be partially explained by the ability of the E2 oncoprotein to induce overexpression of p21 and downregulation of E7, resulting in increased levels of hypophosphorylated pRb protein and ultimately to G1 cell cycle arrest (Ref. 101). The nasopharyngeal carcinoma cell line CNE2 showed around 56 and 79% IR-induced cellular senescence 72 h after 6 and 10 Gy X-ray exposure, respectively (Ref. 83). Since CNE2 cells are p16-deficient, only p53 and p21 protein levels were elevated together with the anti-apoptotic protein Bcl-xL, suggesting senescence was preferred over apoptosis as the cell death mechanism. Conversely, it has been shown that only 5% of p53-deficient HNSCC cancer cells undergo senescence after 4 Gy X-rays radiation treatment, whilst p53-wild type containing cells reach up to 60% senescence-associated *β*-galactosidase-positive cells (Ref. 102). This confirms the prominent role of p53 in driving senescence compared to p16. This confirmed previous observations in 38 different HNSCC cell lines where p53 mutation significantly decreased IR-induced senescence and conferred radioresistance (Ref. 103). Furthermore, the importance of the p53–p21 interaction for the initiation of the senescence pathway has been highlighted (Refs 102, 103).

HNSCC cells exposed to 1 Gy *γ*-radiation have been shown not to display any significant induction of mitotic catastrophe (Ref. 104), whilst a higher dose (6 Gy) of X-rays caused 20% of FaDu cells to undergo this process (Ref. 84) (summarised in Table 1). The increase in mitotic catastrophe induction appears to be p53-independent, in line with previously reported evidence for HCT116 colorectal cancer cell lines after 4 Gy X-ray treatment (Ref. 105). Oesophageal squamous cell carcinoma cell lines have demonstrated a modest increase in mitotic catastrophe markers after 6 Gy X-ray irradiation (Ref. 85), while nasopharyngeal carcinoma cells displayed around 50% of cells undergoing mitotic catastrophe after 20 Gy of X-ray treatment (Ref. 86). Whilst the dose used is different, this could suggest that triggering of mitotic catastrophe as a cell death mechanism could be tumour cell-dependent. Mitotic catastrophe was observed to be the dominant mechanism of cell death in HPV-positive and HPV-negative HNSCC cell lines after a single 4 Gy X-ray radiation exposure, with no major difference between 4 and 72 h timepoint (Ref. 77). Moreover, both types of cell lines tested responded in a similar manner, suggesting that HPV infection has no dramatic impact on the degree of mitotic catastrophe induction.

In summary, evidence suggests that low-LET radiation exposure may trigger different types of HNSCC cell death mechanism, and that the cells undergo a specific pathway depending on the radiation dose but also the cellular genetic profile.

### Cell death mechanisms after high LET exposure

Mechanistic modelling has suggested that high-LET radiation, such as *α*-particles and heavy ions, can cause up to 90% of CDD within the total amount of DNA lesions induced, compared to ~30% in the case of low-LET radiation exposure (Refs 106, 107). CDD has been clearly demonstrated to be a challenge to the DNA repair machinery and can persist in cells and tissues several (6–24) hours after IR, while simple SSBs and DSBs resolve in less than 30 min and 2 h, respectively (Refs 64, 108–111). High-LET radiation can therefore kill cancer cells more efficiently compared to low LET treatments. For example, and as reported for SQ20B laryngeal squamous cells, exposure to 4 Gy low-LET X-rays produced 56% cell survival whereas high-LET carbon ion (LET = 184 keV/μm) at the same dose yielded only 9% of cells surviving (Ref. 112) (Table 2). A reduction in cell survival from 55 to 15% was also observed in UMSCC74A after 4 Gy high-LET proton treatment (LET = 12 keV/μm) compared to 4 Gy low-LET proton exposure (LET = 1 keV/μm), while UMSCC6 cells displayed a less marked difference (35% cell survival for low-LET versus 20% for high-LET) (Refs 64, 113). This demonstrates the differences in inherent radioresistance of the different HNSCC cells to both low and high-LET radiation. Nevertheless, CDD formation is likely the major contributor to this observed reduced survival in response to high-LET radiation thereby triggering apoptosis or mitotic catastrophe, although other mechanisms in cell death pathway activation are likely to be involved once the cell fails to resolve the damage. For example, effects on centrosome biology in HNSCC cell lines have recently been shown in response to high-LET protons (Ref. 113).

**Table 2.** Biological effects of high-LET radiation on head and neck cancer cells

Cancer cell	Dose	Response compared to control	Reference
SQ20B (larynx)	4 Gy high-LET carbon ion (LET = 184 keV/μm)	9% of cells surviving	(Ref. 112)
UMSCC74A (oropharynx)	4 Gy high-LET proton treatment (LET = 12 keV/μm) or low-LET proton exposure (LET = 1 keV/μm)	Reduction in cell survival from 55% (low-LET) to 15% (high-LET)	(Refs 64, 113)
UMSCC6 (oropharynx)	4 Gy high-LET proton treatment (LET = 12 keV/μm) or low-LET proton exposure (LET = 1 keV/μm)	Reduction in cell survival from 35% (low-LET) to 20% (high-LET)	(Ref. 64)
Human tongue carcinoma cells	5 Gy high-LET carbon ion exposure (LET = 70 keV/μm)	5% increase in apoptotic marker	(Ref. 114)
Human tongue carcinoma cells	1 Gy high-LET carbon ion exposure (LET = 70 keV/μm)	No apoptotic response	(Ref. 115)
Human oesophageal carcinoma cells	1–3 Gy high-LET oxygen ion exposure (LET = 154 keV/μm)	Increased apoptotic response	(Ref. 116)

Interestingly, the mechanisms of cell death in response to high-LET radiation in HNSCC cells have not been studied extensively. Human tongue carcinoma cells have been shown to display less than a 5% increase in apoptotic marker activation after 5 Gy high-LET carbon ion exposure (LET = 70 keV/μm) compared to X-ray radiation (Ref. 114), although this was not apparent when the dose was reduced to 1 Gy (Ref. 115) (Table 2). Apoptosis activation has also been reported in a study on radiosensitive human oesophageal carcinoma cells, where caspase 3 activation was 1.8-fold higher after heavy ion irradiation versus X-rays, while only marginal differences were observed in their radioresistant cell counterparts (Ref. 116). This suggests that the cell-killing effect is cell line and radiation quality-dependent, and that perhaps other types of cell death may play a more prominent role. This confirms previous observations in radiosensitive laryngeal squamous cancer cells and their appropriate radioresistant cell lines (Ref. 117). Considering the significant increased therapeutic use of PBT, but also heavy ion radiotherapy, further and more extensive studies are needed to fully elucidate the cell death mechanisms activated in specific HNSCC cell types in response to high-LET radiation exposure.

### Therapeutic strategies

Targeting key proteins involved in cell death mechanisms is a feasible approach in increasing HNSCC radiosensitivity, which can lead to more effective treatment of the tumour. Some targets involved in cell death pathway activation and/or execution have been already identified and explored *in vitro*. In most cases, tumour cells highly express anti-apoptotic proteins, therefore their inhibition could potentially increase cellular radiosensitivity. Survivin is a protein inhibitor of the terminal effector caspases and is highly expressed in several types of cancer, including head and neck malignancies (Refs 118, 119). Efficient downregulation of survivin has been extensively studied with promising results in terms of enhancing tumour sensitivity to various therapeutic interventions (reviewed in (Ref. 120)). Livin, another member of the apoptosis inhibitor protein family, has been found to be associated with tumour progression and poor prognosis in various human cancers. Its inhibition has been shown to suppress tumour cell invasion and enhance cell apoptosis, with elevated expression levels of cleaved caspases 3 and 7 and cleaved PARP in anaplastic thyroid cancer (Ref. 71) and in laryngohypopharyngeal cancer models (Ref. 121). Other anti-apoptotic proteins, including Bcl-2 and vitronectin, have been investigated in several HNSCC subtypes, with their inhibition significantly increasing apoptosis in response to X-rays (Refs 122–124) or *γ*-radiation (Ref. 125). A phase I/II clinical trial initiated to investigate mitochondria-derived activator of caspases as a mimetic therapy in patients with previously untreated stage III/IV HNSCC in combination with cisplatin and radiation is currently ongoing (ClinicalTrials.gov identifier NCT02022098).

Post-radiotherapy, necrosis usually occurs in the oral cavity, maxilla, mandible and salivary glands more than the tumour itself, and the aim therefore is to prevent this to improve patient quality of life. Although several therapies have been reported, there is not a universally accepted approach to tackle necrosis and the treatment options are variable. Surgical treatments are still of preference, but recently some pharmacological options have been investigated, including treatments with antioxidants or biological molecules, such as basic fibroblast growth factor or bone morphogenetic protein-1 (reviewed in (Refs 126, 127)). Nevertheless, inducing necrosis in HNSCC cells is a possibility that has been partially explored. The monoclonal anti-IGF-1R antibody A12 in combination with *γ*-rays has been shown to dramatically induce necrotic death in FaDu-derived xenografts compared to unirradiated controls (Ref. 128), while the second mitochondria-derived activator of caspase mimetic birinapant has proven effective both in caspase-8-deficient and FADD-overexpressing HNSCC cells, inducing programmed necrosis and increasing cell radiosensitivity after X-ray treatment (Refs 129, 130).

Although ATG proteins are often upregulated in tumour cells, the activation of this specific pathway could lead to massive cell death after radiotherapy. The phosphoinositide 3-kinase (PI3K)/protein kinase B (AKT)/mammalian target of rapamycin (mTOR) pathway is a critical regulator of autophagy, and targeting this could be an important therapeutic strategy enhancing the sensitivity of tumour cells to IR. Several pharmacological inhibitors have been tested on various cancer cell types, including HNSCC cells, both *in vitro* and in xenograft models with promising results (reviewed in (Ref. 131)). A phase I clinical trial is currently ongoing in high-risk patients with locally advanced squamous cell carcinoma to test a potent and highly specific oral pan-class I PI3K inhibitor, BKM120 (ClinicalTrials.gov Identifier NCT02113878). Other ATG targets have been investigated, including Kelch-like ECH-associated protein 1 (Ref. 80) and p62, whose overexpression has been reported to induce autophagy in HNSCC cells (Ref. 132).

The induction of senescence can be clinically beneficial, but currently there are no specific senescence-inducing agents available. The ability of cells to enter radiation-induced senescence is almost strictly linked to p53. In fact, it has been reported that HNSCC cells with wild-type or non-disruptive mutations of p53 undergo senescence after radiotherapy, while p53 mutant cells are more radioresistant and show higher levels of senescence markers after treatment with metformin (anti-diabetic agent that has been shown to induce reactive oxygen species) (Ref. 103). Reactivation of p53 restored senescence and increased radiosensitivity in p53 mutant HNSCC cells, although interestingly the study reported that the mechanism is only partially p53-dependent (Ref. 133). Treatment with rapamycin or the mTOR inhibitor PP242 in parotid carcinoma cells *in vitro* increased heterochromatin formation, and induced irreversible growth arrest and premature senescence. Whilst in tumour xenografts, PP242 delayed tumour regrowth after X-ray irradiation and increased senescence-associated *β*-galactosidase staining (Ref. 134).

Inhibition of proteins involved in DNA replication and mitosis could be a valid therapeutic solution to force the cells into mis-segregation of chromosomes and promote aberrant mitotic division. As mitotic catastrophe is a relatively new cellular endpoint, the current literature is scarce and only a few candidates have been investigated as chemotherapeutic targets. Polo-like kinase 1 (PLK1) is a serine/threonine kinase which functions as a pleiotropic master regulator of mitosis and regulates DNA replication after stress (Ref. 135). PLK1 depletion has been reported to induce mitotic cell cycle arrest and inhibit the separation of sister chromatids in oesophageal cancer cells, causing failure of cytokinesis followed by massive apoptotic cell death (Ref. 136). These results have been confirmed in human nasopharyngeal cancer cells, where PDK1 inhibition was found to greatly reduce cell survival, alone or in combination with radiation, due to G2/M cell cycle arrest and aberrant spindle formation, which in turn caused mitotic catastrophe (Ref. 137). Furthermore, co-treatment with PLK1 inhibitor and inhibitors targeting Aurora A and Aurora B enhanced metaphase arrest and mitotic slippage in nasopharyngeal cancer cells, ultimately inducing mitotic catastrophe (Ref. 138). The Aurora A and B protein kinases are key players in mitotic control and therefore another set of potential targets to increase cancer radiosensitivity. Aurora B inhibition in fact has been demonstrated to lead to G2/M accumulation, polyploidy and subsequent cell death by mitotic catastrophe in anaplastic thyroid carcinoma *in vitro* and reduced growth of ATC tumour xenogratfs (Ref. 139). Aurora A depletion appears to have a limited effect on HPV-negative HNSCC cells when administered alone, although still causing spindle defects and cytostasis, while co-treatment with a WEE1 cell cycle checkpoint kinase inhibitor triggered mitotic catastrophe *in vitro* and reduced tumour growth in FaDu and Detroit 562-derived xenografts (Ref. 140). Other potential cellular targets for inhibitors that have yielded promising results as chemotherapeutic agents against HNSCC cells include WEE1 (Refs 141–143), CHK1/2 (Refs 141, 143) and PP2A (Ref. 86), although further studies are needed in order to determine their specific potential as radiosensitisers.

## Conclusions

Conventional photon (X-ray) radiotherapy has been used for several decades in the treatment of HNSCC, while particle beam therapy including protons and carbon ions have only recently gained increasing utility but which benefit from their high energy localised deposition coupled with the preservation of the surrounding healthy tissue. Nevertheless, current knowledge regarding the molecular mechanisms involved in cancer cell death in response to high-LET radiation compared to conventional low-LET photon therapy is still quite limited, and requires further investigation. Elucidating the different cell death mechanisms activated by tumour cells after high-LET radiotherapy would allow a more targeted therapeutic strategy, with the use of inhibitors specifically designed for proteins involved in enhancing the determined cell death pathway. This will ultimately lead to a more personalised and effective approach during radiotherapy, whilst also enabling a reduction in the dose of radiation needed to obtain a full tumour eradication and therefore limiting the possibility of acute and long-term adverse side-effects associated with irradiation of the associated normal tissues.
